# Effects of Symmetry and Apparent Distance in a Parasagittal-Mirror Variant of the Rubber Hand Illusion Paradigm

**DOI:** 10.3389/fnhum.2021.718177

**Published:** 2021-09-16

**Authors:** Jhana de Silva, Haiwen Chen, Sasha Isaac, Rebekah C. White, Martin Davies, Anne M. Aimola Davies

**Affiliations:** ^1^Research School of Psychology, Australian National University, Canberra, ACT, Australia; ^2^Department of Experimental Psychology, Medical Sciences Division, University of Oxford, Oxford, United Kingdom; ^3^Corpus Christi College, University of Oxford, Oxford, United Kingdom; ^4^Philosophy Department, Monash University, Clayton, VIC, Australia

**Keywords:** body ownership, distance, mirror box, multisensory integration, parasagittal mirror, peripersonal space, rubber hand illusion, symmetry

## Abstract

When I see my face in a mirror, its apparent position (behind the glass) is not one that my own face could be in. I accept the face I see as my own because I have an implicit understanding of how mirrors work. The situation is different if I look at the reflection of my right hand in a parasagittal mirror (parallel to body midline) when my left hand is hidden behind the mirror. It is as if I were looking through a window at my own left hand. The experience of body ownership has been investigated using rubber hand illusion (RHI) paradigms, and several studies have demonstrated ownership of a rubber hand viewed in a frontal mirror. Our “proof of concept” study was the first to combine use of a parasagittal mirror and synchronous stroking of both a prosthetic hand (viewed in the mirror) and the participant’s hand, with a manipulation of distance between the hands. The strength of the RHI elicited by our parasagittal-mirror paradigm depended not on physical distance between the hands (30, 45, or 60 cm) but on apparent distance between the prosthetic hand (viewed in the mirror) and the participant’s hand. This apparent distance was reduced to zero when the prosthetic hand and participant’s hand were arranged symmetrically (e.g., 30 cm in front of and behind the mirror). Thus, the parasagittal-mirror paradigm may provide a distinctive way to assess whether competition for ownership depends on spatial separation between the prosthetic hand and the participant’s hand.

## Introduction

Looking at oneself in a mirror is an everyday example of altering bodily self-awareness. One’s seen body and felt body no longer coincide in space. If I sit in front of a mirror and look at the reflection of my face, then the apparent position of the face that I see is behind the glass and the apparent orientation is toward me. No face that was really in that position and orientation could possibly be my own face. Nevertheless, around 18 months of age, we come to recognize the baby seen in the mirror as ourself (Amsterdam, 1972; Brooks-Gunn and Lewis, 1984).

Over time, ownership of our mirror image becomes automatic under normal conditions, though the sense of ownership is disrupted if, for example, the facial movements seen in the mirror are not synchronous with our active movements of the face (O’Sullivan et al., 2018). This ownership of the mirror image is not just a matter of recognizing oneself, as in a (mirror-reversed) photograph. We learn to use our reflection to guide actions such as combing our hair or adjusting our clothing. More generally, we learn to transform the visual information about apparent position and orientation, so that we can act fluently in mirrored-space – although incorrect beliefs about mirror reflections are widespread (Lawson and Bertamini, 2006).

As we age, mirrors can sometimes re-emerge as a challenge. Some patients with focal onset dementia actually believe that the person they see in the mirror is not them (mirrored-self misidentification; Breen et al., 2000, 2001). Thus, just as the way we view and interact with our environment may alter our perception of that environment (DiZio et al., 1993), so too may viewing and interacting with our body seen in a mirror – in varying ways across the lifespan – modulate our sense of body ownership.

The experience of body ownership has been investigated using the rubber hand illusion (RHI; Botvinick and Cohen, 1998). In the Classic-RHI paradigm, the participant places their left hand out of view, hidden behind an opaque divider, and is asked to look at a rubber left hand positioned in front of them and oriented *egocentrically* (i.e., with the fingers pointing away from them). The participant is able to *see* brush strokes on the rubber hand and able to *feel* (but not see) strokes on their own hand. When the seen strokes are synchronous with the felt strokes, the RHI is elicited. The participant reports their experience of the RHI, by rating their agreement with statements expressing three aspects of the illusion: *ownership*, *causation*, and *visual capture of touch* (VCT). The experimenter may also measure the *proprioceptive drift* of the hidden hand toward the seen rubber hand, by asking the participant to indicate the felt position of their hidden left hand before, and again after, stroking.

In RHI studies by Bertamini et al. (2011) and Kontaris and Downing (2011), participants either viewed a rubber hand directly (as in the Classic-RHI paradigm) or looked at the reflection of a rubber hand in a mirror in front of them (with the direct view of the rubber hand occluded). In the mirror-view condition, the apparent position of the seen rubber hand was behind the glass and oriented *allocentrically* (i.e., with the fingers pointing toward the participant). Bertamini et al. found that the RHI was just as strong (assessed by illusion ratings and proprioceptive drift) in the mirror-view condition as in the direct-view condition; and this finding (for illusion ratings) was replicated by Jenkinson et al. (2013). Kontaris and Downing also added an orientation manipulation, with the fingers of the rubber hand oriented egocentrically or allocentrically. They found that, in the direct-view condition, the RHI (assessed by illusion ratings and proprioceptive drift) was abolished in the allocentric-orientation condition, replicating earlier findings (Ehrsson et al., 2004; also see Jenkinson and Preston, 2015). In contrast, in the mirror-view condition, the RHI was elicited in both orientation conditions – though at a somewhat reduced level compared with direct viewing of a rubber hand oriented egocentrically. Using the moving RHI paradigm (Kalckert and Ehrsson, 2012), Jenkinson and Preston (2015) found *higher* ratings for the illusion of ownership of the rubber hand in mirror-view than in direct-view conditions.

When I see my hand or face in a mirror in front of me, its apparent position (behind the glass) is not one that my own hand or face could be in. I accept it as my own hand or face because I have an implicit understanding of how mirrors work. The situation is different when the mirror is placed in a parasagittal plane (e.g., to the left of body midline). If I sit with my right hand at midline and look to the left into the parasagittal mirror, then the hand seen in the mirror appears to be a left hand behind the glass. No such hand in that position could be my own right hand but it could be my own left hand. For a participant looking at the reflection of a right hand in the parasagittal mirror, it is as if they were looking through a window at their real left hand. Thus, when using a parasagittal mirror, the reflection in the mirror of a real (or rubber) *right* hand can be “superimposed” on a hidden *left* hand behind the mirror.

This specular superimposition has been used to “resurrect” a phantom limb in patients following upper-limb amputation and, in some patients, to relieve pain in the phantom limb (Ramachandran et al., 1995; Ramachandran and Rogers-Ramachandran, 1996). Parasagittal mirrors have also been used to: manipulate the visually perceived distance between participants’ hands (Gallace and Spence, 2005); assess the influence of vision on proprioception (Holmes et al., 2006); investigate visual enhancement of touch (Ro et al., 2004; Longo et al., 2008a); compare tactile illusions in amputees’ phantom limbs and healthy individuals’ intact but untouched limbs (Giummarra et al., 2010); explore relationships between the illusion of ownership, proprioceptive drift, estimates of the hardness of a foam pad, and skin temperature on the hands (Sadibolova and Longo, 2014; Medina et al., 2015; Katsuyama et al., 2018; Crivelli et al., 2021).

For the Parasagittal-Mirror-RHI paradigm used in the present study, the participant placed their left hand out of view, hidden behind the non-reflective side of the parasagittal mirror, and was asked to look *in the mirror* at the reflection of a prosthetic right hand – which appeared as a left hand behind the glass (The participant’s real right hand, which was not relevant to the paradigm, remained in their lap.) When the prosthetic hand and the participant’s hidden hand were stroked synchronously, the participant reported their experience of the RHI by rating their agreement with three illusion statements: *ownership*, *causation*, and *VCT*.

Physical distance between the prosthetic hand and the participant’s hidden hand, and symmetry of the two hands in front of and behind the mirror, were manipulated so that in symmetrical conditions the reflection of the prosthetic right hand was “superimposed” on the participant’s hidden left hand. In two experiments, we used the Classic-RHI paradigm and the Parasagittal-Mirror-RHI paradigm to investigate the experience of the RHI.

Our first research question concerned the strength of the RHI in the parasagittal mirror. We predicted effects for

• Paradigm: The Parasagittal-Mirror-RHI paradigm would elicit higher illusion ratings than the Classic-RHI paradigm with a matched symmetrical arrangement of the prosthetic hand and the participant’s hand in front of and behind the mirror or opaque divider.

Our second research question concerned the effects of distance and symmetry in the Parasagittal-Mirror-RHI paradigm. We predicted that the way in which the physical distance between the prosthetic hand and the participant’s hand was divided either side of the mirror (prosthetic hand **X cm** in front of the mirror + participant’s hand **Y cm** behind the mirror) would be critical. Specifically, we predicted effects for

• Symmetry: Higher illusion ratings would be elicited when the prosthetic hand and the participant’s hand were positioned symmetrically either side of the mirror, even if the physical distance between the prosthetic hand and the participant’s hand differed between 30 cm (15 cm + 15 cm) and 60 cm (30 cm + 30 cm); and
• Asymmetry: Lower illusion ratings would be elicited when the prosthetic hand and the participant’s hand were positioned asymmetrically either side of the mirror, even if the physical distance between the prosthetic hand and the participant’s hand remained constant at 60 cm (15 cm + 45 cm and 30 cm + 30 cm).

## Methods

### Participants

Participants were recruited from the Australian National University, and received a small remuneration. Participants provided informed written consent in accordance with the ethics protocol (2015/397) approved by the Australian National University Research Ethics Committee.

**FIGURE 1:**
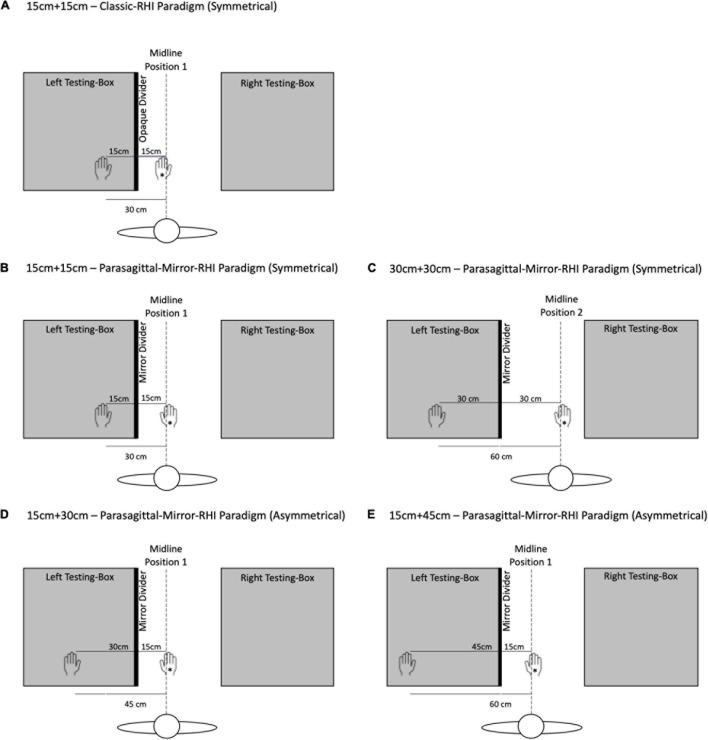
Rubber hand illusion testing unit for the symmetrical placement of the prosthetic hand and the participant’s hidden hand in the Classic-RHI paradigm, and the symmetrical and asymmetrical placements in the Parasagittal-Mirror-RHI paradigm. **(A)** Depicts the testing unit for the Classic-RHI paradigm (participants instructed to look at the index finger of the prosthetic hand), and **(B–E)** depict the testing unit for the Parasagittal-Mirror-RHI paradigm (participants instructed to look at the mirror reflection of the index finger of the prosthetic hand). In Experiment 1, Symmetrical condition **(A,B)**, the total distance between the prosthetic hand and participant’s hand was 30 cm (divided 15 cm + 15 cm in front of and behind the opaque divider or mirror). In Experiment 2, (i) Symmetrical conditions **(B,C)**, the total distance between the prosthetic hand and participant’s hand was either **(B)** 30 cm (divided 15 cm + 15 cm in front of and behind the mirror) or **(C)** 60 cm (divided 30 cm + 30 cm in front of and behind the mirror); (ii) Asymmetrical conditions **(D,E)**, the total distance between the prosthetic hand and participant’s hand was either **(D)** 45 cm (divided 15 cm + 30 cm in front of and behind the mirror) or **(E)** 60 cm (divided 15 cm + 45 cm in front of and behind the mirror). In all conditions, the prosthetic hand, which is marked in the figure with an asterisk (*), was placed at the participant’s midline (between the two boxes), and the participant’s left hand was placed inside the left box (to the left of the participant’s midline), at 15, 30, or 45 cm behind the opaque divider or mirror. There were two midline positions: Midline Position 1 (prosthetic hand positioned 15 cm in front of the mirror) and Midline Position 2 (prosthetic hand positioned 30 cm in front of the mirror).

In Experiment 1, 21 participants (Mean age: 25.6 years, SD: 8.67) were tested with the Classic-RHI paradigm (in which participants were instructed to look directly at the prosthetic hand) and the Parasagittal-Mirror-RHI paradigm (in which direct viewing of the prosthetic hand was not occluded but participants were instructed to look at the mirror reflection of the prosthetic hand). In both paradigms, the physical distance between the prosthetic hand and the participant’s hand was 30 cm, divided symmetrically: 15 cm + 15 cm in front of and behind the opaque divider or mirror (see Figures 1A,B).

In Experiment 2, 24 new participants (Mean age: 23 years, SD: 1.49) were tested only with the Parasagittal-Mirror-RHI paradigm. There were two symmetrical conditions (physical distance divided 15 cm + 15 cm or 30 cm + 30 cm in front of and behind the mirror) and two asymmetrical conditions (15 cm + 30 cm or 15 cm + 45 cm in front of and behind the mirror), which resulted in three different physical distances (30, 45, and 60 cm) between the hands (see Figures 1B–E).

### Apparatus and Procedure

The custom-built RHI testing unit consisted of two boxes – each 70 cm (L) × 70 cm (W) × 30 cm (H) – with a lid that slid across the top of the boxes and served to keep the prosthetic hand hidden from the participant’s view between trials. A left prosthetic hand was used for the Classic-RHI paradigm and a right prosthetic hand was used for the Parasagittal-Mirror-RHI paradigm (because, when viewed in the mirror, a right prosthetic hand appears as a left prosthetic hand). In both experiments, the prosthetic hand was placed in the gap between the two boxes, and aligned with the participant’s midline. In the Parasagittal-Mirror-RHI paradigm, a 70 cm (L) × 30 cm (H) mirror was attached to the outside wall of the left testing-box, which allowed the participant to view the reflection of the prosthetic hand. In the Classic-RHI paradigm, the mirror was removed so that the participant saw only the opaque divider (i.e., the wall of the left testing-box).

The participant was seated (across from the experimenter) in front of the testing unit with the box lid closed. The participant was asked to rest their right hand in their lap and place their left hand in the left testing-box. A black barber’s cape was draped around their neck, and was stretched out and attached to both testing boxes to obscure visual feedback from the participant’s body. Before the experiment began, the participant was shown the prosthetic hand and it was demonstrated, first how the index finger of the prosthetic hand, and then how the index finger of their own hand, would be stroked. The participant then practised rating the RHI by responding to nine statements (see Table 1; Botvinick and Cohen, 1998) using a digital touch-screen tablet.

**TABLE 1:** Questionnaire for Assessing the Rubber Hand Illusion in the Classic-RHI Paradigm and the Parasagittal-Mirror-RHI Paradigm: Three Illusion Statements (S1–S3) and Six Control Statements (S4–S9).

**Statement #**	**Illusion versus Control Statements**	**Statements**
S1	Ownership	I felt as if the rubber hand were my hand
S2	Causation	It seemed as though the touch I felt was caused by the paintbrush touching the rubber hand
S3	Visual Capture of Touch	It seemed as if I were feeling the touch of the paintbrush in the location where I saw the rubber hand touched
S4	Control	It felt as if my (real) hand were drifting towards the right (towards the rubber hand)
S5	Control	It seemed as if I might have more than one left hand or arm
S6	Control	It seemed as if the touch I was feeling came from somewhere between my own hand and the rubber hand
S7	Control	It felt as if my (real) hand were turning “rubbery”
S8	Control	It appeared (visually) as if the rubber hand were drifting towards the left (towards my hand)
S9	Control	The rubber hand began to resemble my own (real) hand, in terms of shape, skin tone, freckles or some other visual feature

*Nine statements (three illusion statements and six control statements) were presented in randomized order at the end of each trial. Participants responded on a visual analog scale, with indicative marks at only the two end points of the scale: 0 “Not at all” and 6 “Very strongly agree”. Participants used a slider on a digital touch-screen tablet, with 0 and 6 serving to provide the participant with reference points when selecting the point along the line that best indicated their rating of the RHI. In Experiment 1, participants used a Samsung Galaxy 10 inch Tablet with a stylus, and in Experiment 2, participants used an 8 inch iPad Tablet and their finger.*

Once the participant understood the procedure, the experimenter opened the box lid to reveal the prosthetic hand. A cloth was draped over the base of the prosthetic hand to give the impression that the prosthetic hand was attached to the end of the participant’s arm under the barber’s cape. The experimenter instructed the participant that, for the duration of the trial, they were to look either at: (i) the index finger of the prosthetic hand (Classic-RHI paradigm); or (ii) the mirror reflection of the index finger of the prosthetic hand (Parasagittal-Mirror-RHI paradigm). Two fine-haired paintbrushes were used to stroke both index fingers from the metacarpophalangeal joint to the tip of the finger. Stroking consisted of a random sequence of tapping interspersed with long and short brushstrokes, which were administered at a consistent pressure and speed. Stroking of the prosthetic hand and the participant’s hidden hand could be: (i) synchronous (temporally congruent); or (ii) asynchronous (temporally incongruent). The two stroke types were pseudo-randomized to avoid order effects. Each stroke type was administered twice per participant for each experiment condition. In Experiment 1, there were eight trials (two synchronous and two asynchronous trials for each of the two paradigms: Classic-RHI and Parasagittal-Mirror-RHI) with a stroking duration of 120 s. In Experiment 2, there were 16 trials (two synchronous and two asynchronous trials for each placement of the two hands: 15 cm + 15 cm, 15 cm + 30 cm, 15 cm + 45 cm, 30 cm + 30 cm) with a stroking duration of 90 s.

At the end of each trial, the box lid was closed, and the participant was instructed not to move their left hand, and to use their right hand to respond to the nine statements. When they finished responding, they were asked to remove their left hand from the testing unit and to flex and extend their fingers before beginning the next trial.

### Statistical Analysis Plan

Mixed-effects beta regression with a logit link function was used to analyze the ratings (0–6) for the experiment statements (Illusion, Control) – the continuous doubly-bounded variable. The beta distribution supports continuous variables within the (0,1) range, but is undefined at the boundary values of zero and one; therefore, all of the raw ratings were divided by six and were shrinkage-transformed to move the boundary values slightly away from the boundary. See Equation 1 (Smithson and Shou, 2020, p. 51) for the formula in which N is the sample size.


(1) $$y_{s\,h\,r\,i\,n\,k}=\frac{y\,\left(N-1\right)+0.5}{N}$$

Ratings for each illusion statement (Ownership, Causation, VCT) were analyzed separately with the averaged ratings for the control statements (Averaged-Control ratings). For Experiment 1, three within-subject predictors – Stroke (synchronous, asynchronous); Statement (Ownership/Causation/VCT, Averaged-Control); Paradigm (Classic-RHI, Parasagittal-Mirror-RHI) – were entered as fixed effects and random slopes that captured the dependencies in the repeated-measures design (Barr et al., 2013). For Experiment 2, three within-subject predictors -- Stroke (synchronous, asynchronous); Statement (Ownership/Causation/VCT, Averaged-Control); Distance (15 cm + 15 cm, 15 cm + 30 cm, 15 cm + 45 cm, 30 cm + 30 cm) -- were entered as fixed effects, but only Stroke and Statement were entered as random slopes^1^. For both experiments, Participant was entered as the random intercept.

All analyses were carried out in R (version 4.0.5) with the “*glmmTMB*” package for mixed-effects beta regression, “*car*” package for Type-III analysis-of-variance tables with Wald chi-square tests, and “*emmeans*” package for *post hoc* pairwise comparisons with Tukey-corrections for *p*-values.

## Results

### Stroke and Statement Effects

For both experiments, there were significant main effects for Stroke and Statement, and a significant Stroke × Statement interaction. *Post hoc* pairwise comparisons indicated there were higher ratings for synchronous compared with asynchronous stroking for each of the illusion statements (Ownership, Causation, VCT). There were no synchronous versus asynchronous differences for the Averaged-Control ratings.

**FIGURE 2:**
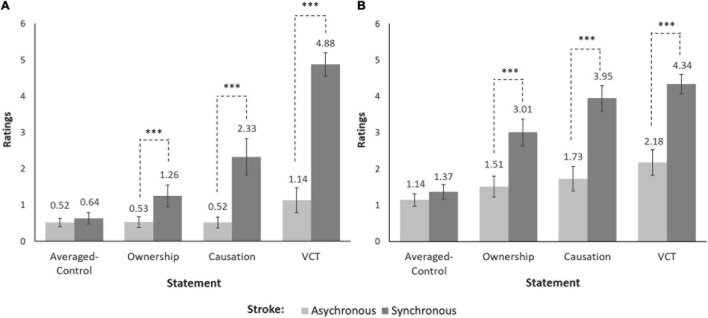
Stroke and Statement effects for Experiment 1 and Experiment 2. The back-transformed means and within-subject standard errors for illusion ratings (Ownership, Causation, VCT) and Averaged-Control ratings are illustrated for Experiment 1 **(A)** and Experiment 2 **(B)**. Separate analyses of ratings for each illusion statement (Ownership, Causation, VCT) with the Averaged-Control ratings indicated that synchronous stroking elicited significantly higher illusion ratings than asynchronous stroking, whereas the Averaged-Control ratings for synchronous and asynchronous stroking were not significantly different. The estimated marginal means and within-subject standard errors of shrinkage-transformed ratings were obtained from the “*effect*” package in R and were back-transformed to the original scale (0–6). ***p < 0.001.

• Experiment 1 (see Figure 2A)

Main effects: Stroke [Ownership, χ^2^ (1) = 15.17, *p* < 0.001; Causation, χ^2^ (1) = 23.95, *p* < 0.001; VCT, χ^2^ (1) = 63.10, *p* < 0.001] and Statement [Ownership, χ^2^ (1) = 9.93, *p* = 0.002; Causation, χ^2^ (1) = 15.91, *p* < 0.001; VCT, χ^2^ (1) = 43.85, *p* < 0.001].

Stroke × Statement interaction [Ownership, χ^2^ (1) = 17.36, *p* < 0.001; Causation, χ^2^ (1) = 65.15, *p* < 0.001; VCT, χ^2^ (1) = 145.15, *p* < 0.001].

*Post hoc* pairwise comparisons for synchronous versus asynchronous stroking: Illusion ratings (Ownership, *b* = 0.86, 95% CI [0.46, 1.27], *t*(316) = 5.53, *p* < 0.001; Causation, *b* = 1.70, 95% CI [1.15, 2.24], *t*(316) = 8.00, *p* < 0.001; VCT, *b* = 2.74, 95% CI [2.17, 3.30], *t*(316) = 12.57, *p* < 0.001) and Averaged-Control ratings (all *p*s ≥ 0.53).

• Experiment 2 (see Figure 2B)

Main effects: Stroke [Ownership, χ^2^ (1) = 24.70, *p* < 0.001; Causation, χ^2^ (1) = 32.46, *p* < 0.001; VCT, χ^2^ (1) = 27.02, *p* < 0.001] and Statement [Ownership, χ^2^ (1) = 32.22, *p* < 0.001; Causation, χ^2^ (1) = 23.85, *p* < 0.001; VCT, χ^2^ (1) = 42.26, *p* < 0.001].

Stroke × Statement interaction [Ownership, χ^2^ (1) = 56.35, *p* < 0.001; Causation, χ^2^ (1) = 115.79, *p* < 0.001; VCT, χ^2^ (1) = 108.07, *p* < 0.001].

*Post hoc* pairwise comparisons for synchronous versus asynchronous stroking: Illusion ratings (Ownership, *b* = 1.04, 95% CI [0.69, 1.39], *t*(745) = 7.60, *p* < 0.001; Causation, *b* = 1.49, 95% CI [1.07, 1.90], *t*(745) = 9.23, *p* < 0.001; VCT, *b* = 1.46, 95% CI [1.01, 1.90], *t*(745) = 8.49, *p* < 0.001) and Averaged-Control ratings (all *p*s ≥ 0.43).

### Research Question 1. Paradigm Effects – Classic-RHI Paradigm and Parasagittal-Mirror-RHI Paradigm

**FIGURE 3:**
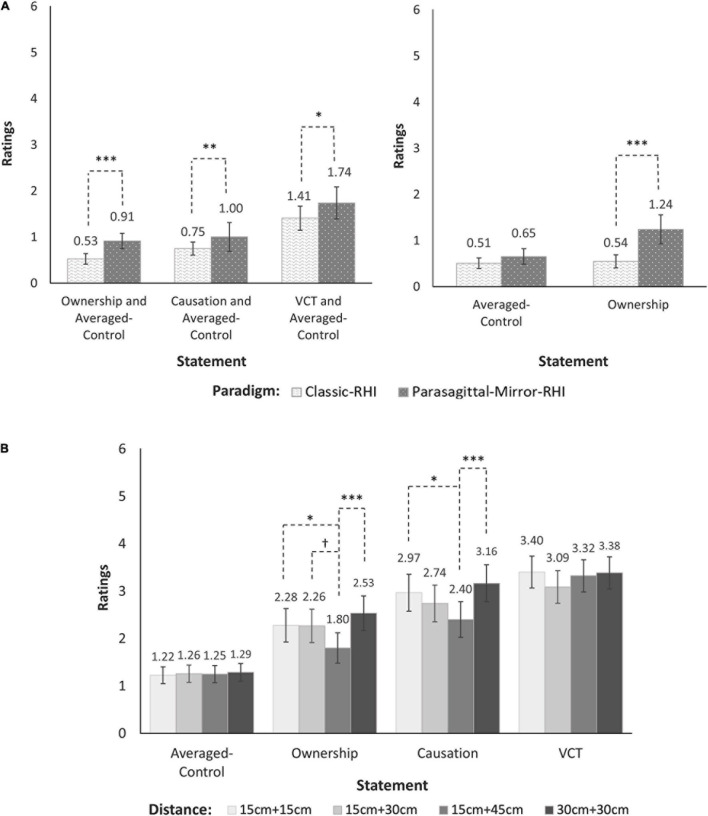
Paradigm effects (Experiment 1), and symmetry *versus* asymmetry effects in four distance conditions (Experiment 2). The back-transformed means and within-subject standard errors for illusion ratings (Ownership, Causation, VCT) and Averaged-Control ratings are illustrated for **(A)** the effects of Paradigm (Experiment 1) and **(B)** the effects of Distance (Experiment 2). For Experiment 1 **(A)**, separate analyses of ratings for each illusion statement (Ownership, Causation, VCT) with the Averaged-Control ratings demonstrated significant main effects for Paradigm (left side) and, for the Ownership-illusion statement, there was a significant Paradigm × Statement interaction (right side). This interaction indicated that the Ownership-illusion ratings were significantly higher for the Parasagittal-Mirror-RHI paradigm compared with the Classic-RHI paradigm, whereas there was no significant difference between paradigms for the Averaged-Control ratings. For Experiment 2 **(B)**, separate analyses of ratings for each illusion statement (Ownership, Causation, VCT) with the Averaged-Control ratings demonstrated significant main effects for Distance, and significant Distance x Statement interactions for the Ownership-illusion and Causation-illusion statements, but not for the VCT-illusion statement. These interactions indicated there were no significant differences in illusion ratings between the two Symmetrical conditions (15 cm + 15 cm versus 30 cm + 30 cm), and there were higher illusion ratings for each of these symmetrical conditions compared with the asymmetrical 15 cm + 45 cm condition. The estimated marginal means and within-subject standard errors of shrinkage-transformed ratings were obtained from the “*effect*” package in R and were back-transformed to the original scale (0–6). *p < 0.05, **p < 0.01, ***p < 0.001, and ^†^*p* = 0.0502.

For Experiment 1 (see Figure 3A), there was a significant main effect for Paradigm (Classic-RHI, Parasagittal-Mirror-RHI) for each of the illusion statements (Ownership, Causation, VCT). For Causation and VCT, these main effects indicated there were higher overall ratings for the Parasagittal-Mirror-RHI paradigm compared with the Classic-RHI paradigm.

• Main effect: Paradigm [Ownership, χ^2^ (1) = 12.93, *p* < 0.001; Causation, χ^2^ (1) = 7.65, *p* = 0.006; VCT, χ^2^ (1) = 5.09, *p* = 0.02].
• Paradigm × Statement interaction [Ownership, χ^2^ (1) = 13.48, *p* < 0.001]. There were no other significant two- or three-way interactions with Paradigm for the three illusion statements (all *p*s ≥ 0.13).

*Post hoc* pairwise comparisons indicated that for Ownership there were higher illusion ratings for the Parasagittal-Mirror-RHI paradigm compared with the Classic-RHI paradigm, *b* = 0.83, 95% CI [0.40, 1.26], *t*(316) = 4.98, *p* < 0.001, but there were no paradigm differences for the Averaged-Control ratings, *p* = 0.54.

### Research Question 2. Symmetry and Asymmetry Effects in Four Distance Conditions

For Experiment 2 (see Figure 3B), there was a significant main effect for Distance (15 cm + 15 cm, 15 cm + 30 cm, 15 cm + 45 cm, 30 cm + 30 cm), and a significant Distance × Statement interaction for Ownership and Causation, but not for VCT.

• Main effect: Distance [Ownership, χ^2^ (3) = 12.80, *p* = 0.005; Causation, χ^2^ (3) = 11.26, *p* = 0.01; VCT, *p* = 0.57].
• Distance × Statement interaction [Ownership, χ^2^ (3) = 10.46, *p* = 0.02; Causation, χ^2^ (3) = 9.81, *p* = 0.02; VCT, *p* = 0.48]. There were no other significant two- or three-way interactions with Distance for the three illusion statements (all *p*s ≥ 0.32).

*Post hoc* pairwise comparisons indicated that for Ownership and Causation there were no significant differences in illusion ratings between the two symmetrical conditions (15 cm + 15 cm versus 30 cm + 30 cm; both *p*s ≥ 0.76), and there were higher illusion ratings for each of these conditions compared with the asymmetrical 15 cm + 45 cm condition:

• 15 cm + 15 cm versus 15 cm + 45 cm (Ownership, *b* = 0.34, 95% CI [0.02, 0.66], *t*(745) = 3.18, *p* = 0.03; Causation, *b* = 0.37, 95% CI [0.03, 0.70], *t*(745) = 3.30, *p* = 0.02);
• 30 cm + 30 cm versus 15 cm + 45 cm (Ownership, *b* = 0.51, 95% CI [0.18, 0.83], *t*(745) = 4.76, *p* < 0.001; Causation, *b* = 0.49, 95% CI [0.15, 0.83], *t*(745) = 4.39, *p* < 0.001).

There were no differences in illusion ratings between other pairs of conditions (all *p*s ≥ 0.21) with one exception that was of interest, the comparison between 15 cm + 30 cm and 15 cm + 45 cm (Ownership, *p* = 0.0502). The Averaged-Control ratings did not differ for any Distance comparisons (all *p*s ≥ 0.99).

## Discussion

Our study is the first, as far as we know, to combine use of a parasagittal mirror, and synchronous stroking of both a prosthetic hand (viewed in the mirror) and the participant’s hand, with a distance manipulation (see Supplementary Material D1). The main aim of the current research was to investigate the Parasagittal-Mirror-RHI paradigm systematically.

First, we demonstrated that the Parasagittal-Mirror-RHI paradigm elicits the RHI. Participants’ ratings for the Ownership-, Causation-, and VCT-illusion statements (but not their Averaged-Control ratings) were higher following synchronous stroking than following asynchronous stroking. Second, we compared the strength of the RHI elicited by the Classic-RHI and Parasagittal-Mirror-RHI paradigms using a matched symmetrical set-up (Experiment 1). The findings supported our prediction, in that the Parasagittal-Mirror-RHI paradigm elicited higher illusion ratings than the Classic-RHI paradigm for the Ownership-illusion statement, and higher overall ratings.

Third, we manipulated distance and symmetry in the Parasagittal-Mirror-RHI paradigm (Experiment 2), and our predictions were supported. In contrast with findings of reduced illusion ratings with increased distance for the Classic-RHI paradigm (Lloyd, 2007; Aimola Davies et al., 2013; Preston, 2013; Kalckert et al., 2019), the distance effect for the Parasagittal-Mirror-RHI paradigm was clearly *not* driven by differences in the *physical* distance between the hands. In the two symmetrical conditions (15 cm + 15 cm, 30 cm + 30 cm), the physical distance between the hands was different (30 cm *versus* 60 cm) while the apparent distance was the same (0 cm) – and the illusion was equally strong. In the symmetrical 30 cm + 30 cm condition compared with the asymmetrical 15 cm + 45 cm condition, the physical distance between the hands was the same (60 cm) while the apparent distance was different (0 cm *versus* 30 cm) – and the Ownership-illusion and Causation-illusion ratings were reduced (though the illusion was not abolished) in the 15 cm + 45 cm condition. Thus, the RHI can be elicited using the Parasagittal-Mirror-RHI paradigm, both when the prosthetic hand (viewed in the mirror) is apparently superimposed on the participant’s hidden hand and when it is apparently separated from the participant’s hand by 15 or 30 cm. The strength of the illusion depends on the apparent distance between the prosthetic hand (viewed in the mirror) and the participant’s hand, rather than on the physical distance between the hands (see Supplementary Material D2).

The Parasagittal-Mirror-RHI paradigm differs from both the Classic-RHI paradigm and the Frontal-Mirror-RHI paradigm, in that it allows the *apparent* position of the prosthetic hand (when viewed in the parasagittal mirror) to coincide with the *physical* position of the participant’s real hand. This superimposition would explain the stronger illusion ratings in the Parasagittal-Mirror-RHI paradigm compared with the Classic-RHI paradigm (Experiment 1) and in the symmetrical conditions compared with the asymmetrical conditions (Experiment 2).

### Future Directions

Many RHI studies collect data on proprioceptive drift as well as illusion ratings but, in our “proof of concept” study, we prioritized illusion ratings. When a parasagittal mirror is used to superimpose the reflection of a prosthetic hand on the participant’s hidden hand, there is a strong illusion of ownership of the prosthetic hand viewed in the mirror, and no scope for proprioceptive drift of the participant’s hand toward the apparent position of the prosthetic hand (Hohwy and Paton, 2010). When a distance manipulation is included, however, there is scope for proprioceptive drift in at least some conditions (see Medina et al., 2015). It would thus be of interest to investigate the relationship between illusion ratings and proprioceptive drift in the Parasagittal-Mirror-RHI paradigm.

It would also be of interest to use illusion statements that reflect loss of the sense of ownership of a body part (“disembodiment”: Longo et al., 2008b; Romano et al., 2021) to investigate whether ownership of a prosthetic hand is accompanied by disownership of the participant’s real hand (Lane et al., 2017). The Parasagittal-Mirror-RHI paradigm may allow us to assess whether competition for ownership between the prosthetic hand and the participant’s hand depends on spatial separation between the hands or results from a “no more than two hands” constraint imposed by a body model (see de Vignemont, 2011).

Finally, and more generally, similarities and differences between parasagittal-mirror viewing and frontal-mirror viewing are not yet fully understood (see Supplementary Materials D3, D4). One of several questions that warrant further investigation is whether images in *parasagittal* mirrors are just as “immediately and effortlessly” related to the objects from which they originate as images in *frontal* mirrors are (Bertamini et al., 2011, p. 1114).

## Data Availability Statement

The original contributions presented in the study are included in the article/Supplementary Material, further inquiries can be directed to the corresponding authors.

## Ethics Statement

The studies involving human participants were reviewed and approved by the Australian National University Human Research Ethics Committee. The participants provided their written informed consent to participate in this study.

## Author Contributions

AA, JS, MD, RW, and SI devised the experiment and contributed to the design of the study. JS and SI collected the data. AA, HC, and JS analyzed the data. AA, JS, and MD drafted the manuscript. All authors contributed to editing and revision of the manuscript and approved the final version for submission.

## Conflict of Interest

The authors declare that the research was conducted in the absence of any commercial or financial relationships that could be construed as a potential conflict of interest.

## Publisher’s Note

All claims expressed in this article are solely those of the authors and do not necessarily represent those of their affiliated organizations, or those of the publisher, the editors and the reviewers. Any product that may be evaluated in this article, or claim that may be made by its manufacturer, is not guaranteed or endorsed by the publisher.

## Footnotes

1 Experiment 2: When Stroke, Statement and Distance were entered as random slopes, the model did not converge (the algorithm failed to reliably detect the maximum of the log-likelihood function); this problem could not be resolved by restarting, increasing iterations, or changing optimizers. In cases of convergence failure, it is sometimes necessary to simplify the random effects structure (Barr et al., 2013). Distance was removed from the random slopes to achieve convergence.
