# Supplementary material for: Effects of Symmetry and Apparent Distance in a Parasagittal-Mirror Variant of the Rubber Hand Illusion Paradigm
Source: Front Hum Neurosci. 2021 Sep 16;15:718177. doi: 10.3389/fnhum.2021.718177 (PMC8481812; doi:10.3389/fnhum.2021.718177)
Supplement: Supplementary file 1 [file Presentation_1.pdf]

Effects of symmetry and apparent distance in a parasagittal-mirror variant of the rubber hand illusion paradigm

JHANA DE SILVA, HAIWEN CHEN, SASHA ISAAC, REBEKAH C. WHITE, MARTIN DAVIES AND ANNE M. AIMOLA DAVIES

Supplementary Material

Discussion

D1. Antecedents of our study

Our study used a prosthetic hand viewed in a parasagittal mirror and stroking was applied to both the prosthetic hand and the participant's hand. The study included a comparison with the no-mirror Classic-RHI paradigm (Experiment 1) and a distance manipulation for the Parasagittal-Mirror-RHI paradigm only (Experiment 2). Our Parasagittal-Mirror-RHI paradigm and our experimental manipulations have partial antecedents in several studies with varying aims and research questions. Here we briefly review five studies in which a parasagittal-mirror paradigm was used and an illusion of ownership or embodiment was assessed with a questionnaire (see Table S1).

In Longo et al.'s (2008a) study, participants looked in a parasagittal mirror at the reflection of a left rubber hand, which was resting palm up in front of the mirror, so that it was superimposed on their real right hand, which was hidden behind the mirror. Synchronous or asynchronous stroking, or no stroking, was applied to the palmar surfaces of the index fingers of both hands (90 s), tactile acuity was assessed, and then participants gave ratings for several illusion statements. Participants whose performance on the tactile acuity task was close to chance showed enhanced acuity when, following synchronous stroking, they felt as though they were looking directly at their own hand. The authors suggested that visual enhancement of touch "is not just an effect of seeing *a* hand, but (at least partly) of seeing *my* hand" (2008a, p. 1188).

Giummarra et al. (2010) used a midsagittal mirror in a modified ('no touch on participant's hand') RHI paradigm. Participants looked in the mirror at the reflection of a rubber hand, superimposed on their real hand, as the rubber hand was stroked, touched, or otherwise manipulated. A substantial proportion of participants reported the illusion of ownership of the rubber hand (viewed in the mirror) and illusory sensations (e.g., of touch) on their real hand behind the mirror, even though their hand was not touched or manipulated in any way. The authors concluded that, in conditions of specular superimposition, "simultaneous stroking or stimulation of the participant's target [real] hand is not necessary to induce illusions of embodiment and corresponding perceptual illusions" (2010, p. 114). White and Aimola Davies (2015) commented:

> Perhaps this is not surprising given that in the Giummarra et al. study it would have seemed to the participant that she was looking at her own left hand through a sheet of glass. (2015, p. 147)

Sadibolova and Longo (2014, Experiment 1) used a midsagittal mirror in a ‘no stroking, view only’ paradigm. Participants looked in the mirror (120 s) at the reflection of their right hand, which was 20 cm in front of the mirror, so that it was superimposed on their left hand, which was 20 cm behind the mirror. During this period, participants felt that they were looking at their own left hand and the temperature of their hidden left hand increased significantly above baseline. In contrast, when participants looked directly at their right hand (‘no-mirror’ condition) there was no such temperature increase in the left hand. This study confirmed that, without any touch or stroking, extended viewing of the reflection of a right hand, whose apparent position coincides with the position of one’s hidden left hand, is sufficient to elicit a strong illusion that one is looking at one’s own left hand.

Medina et al. (2015) used a parasagittal mirror in a tapping paradigm (synchronous, asynchronous, or no movement) with a distance manipulation. Participants tapped (60 s) with their left index finger on the mirror, and with their right index finger on the back of the mirror (so that the reflection of the left hand was superimposed on the hidden right hand) or on a surface 6´´ or 12´´ behind the mirror. There was also a no-mirror condition, in which the mirror was replaced by an opaque divider. Participants gave ratings for illusion statements, and proprioceptive drift of the right hand was also measured. There were effects of paradigm (mirror *versus* no mirror) and of distance (0´´, 6´´, 12´´) on the strength of the illusion and on the magnitude of proprioceptive drift. It is of interest that, in the mirror paradigm and with synchronous tapping, a measure of *proportional* proprioceptive drift in the 6´´ and 12´´ distance conditions (drift toward the mirror as a proportion of the distance from the mirror) was significantly predicted by participants’ ownership illusion ratings.

Finally, in an investigation of the effect of the illusion of ownership on skin temperature, Crivelli et al. (2021) used a version of Medina et al.’s (2015) paradigm with the participant’s two index fingers tapping on mirrors 6´´ apart. Synchronous tapping elicited the illusion of ownership of the seen hand as viewed in the mirror (appearing to be immediately behind the glass) and proprioceptive drift of the hidden hand toward the apparent position of the seen hand. Following synchronous stroking, there was a reduction in the temperature of both hands, and the magnitude of the temperature change was significantly correlated with proprioceptive drift but not with illusion ratings.

Each of these partial antecedents of our study makes a substantial contribution to the investigation of ownership and embodiment using a parasagittal (or midsagittal) mirror. Our study is novel in combining use of a prosthetic hand viewed in a parasagittal mirror, stroking of both the prosthetic hand and the participant’s hand, comparison with a no-mirror paradigm (Experiment 1), and a distance manipulation (Experiment 2).

Table S1

Five Partial Antecedents of Our Study

Study<br>and research question	Rubber hand	Stroking	No-mirror condition	Distance
**The present study (2021)**<br>Effects of paradigm and distance	✓	**Both hands**	✓	✓
Longo et al. (2008a)<br>Visual enhancement of touch	✓	Both hands	✗	✗
Giummarra et al. (2010)<br>Embodiment and illusory sensations	✓	Rubber hand only	✗	✗
Sadibolova and Longo (2014)<br>Ownership and skin temperature	✗	Viewing paradigm	✓	✗
Medina et al. (2015)<br>Embodiment and multisensory integration	✗	Tapping paradigm	✓	✓
Crivelli et al. (2021)<br>Ownership and skin temperature	✗	Tapping paradigm	✗	✗

Note. All five studies used a parasagittal (or midsagittal) mirror and assessed ownership or embodiment with a questionnaire.
Rubber hand: Did the experimental paradigm include a rubber hand?
Stroking: Was stroking applied to both the rubber hand and the participant's hand; or if there was no rubber hand what kind of paradigm was used?
No-mirror condition: Did the study include a comparison between the parasagittal-mirror condition and a no-mirror condition?
Distance: Did the study compare ownership or embodiment ratings in the mirror condition across different distance conditions?

Supplementary Material

Discussion

D2. Distance conditions, physical distance and apparent distance

It is important to distinguish two notions of distance:

the physical distance between the prosthetic hand (positioned at body midline, in front of the mirror) and the participant's hand (hidden behind the mirror)

versus

the apparent distance – the distance between the apparent position of the prosthetic hand (viewed in the mirror) and the position of the participant's hidden hand.

Table S2 shows the physical and apparent distances between the two hands for each of the four distance conditions. When the prosthetic hand and the participant's hand are positioned symmetrically, in front of and behind the mirror, then the apparent distance between the hands is zero (superimposition). When the prosthetic hand and the participant's hand are positioned asymmetrically, in front of and behind the mirror, then the apparent distance between the hands is not zero (separation); but the apparent distance is still less than the physical distance.

Table S2

Physical and Apparent Distance in Four Distance Conditions

**Distance condition**	**15 cm + 15 cm Symmetrical**	**15 cm + 30 cm Asymmetrical**	**15 cm + 45 cm Asymmetrical**	**30 cm + 30 cm Symmetrical**
Physical distance	30 cm	45 cm	60 cm	60 cm
Apparent distance	0 cm	15 cm	30 cm	0 cm

Supplementary Material

Discussion

D3. Stronger illusion in a frontal mirror

When participants look at a rubber hand in a mirror in front of them (frontal-mirror view), the RHI is as strong as (Bertamini et al., 2011) or stronger than (Jenkinson and Preston, 2015) when they look directly at the rubber hand with no mirror (direct view). Similar results for frontal-mirror viewing have been obtained using the full-body illusion (Preston et al., 2015) and self-observation of the body (Jenkinson and Preston, 2017). One aspect of frontal-mirror viewing is that it provides a third-person or allocentric perspective on our own body and (as a matter of optics) objects viewed in a mirror are seen as being behind the glass. Another aspect is that we "immediately and effortlessly relate images in mirrors to the objects from which those images originate" (Bertamini et al., 2011, p. 1114). It seems that, for those who are familiar with frontal mirrors, mirror-reflections are readily – even automatically – interpreted as images of objects in the space in front of the mirror, the space around the viewer (*peripersonal* space). Thus, for example, while looking in a frontal mirror, I reach above and behind my right shoulder to grasp an apple that I see being held there.

Effortless or automatic mapping of objects seen in a frontal mirror, into the space around the viewer in front of the mirror, may be sufficient to explain why the RHI is just as strong in frontal-mirror-view conditions as in direct-view conditions. Jenkinson and Preston (2015) found, however, that a *stronger* illusion of ownership was elicited by frontal-mirror viewing. How might that be explained?

In neural information processing, a comparator signals a prediction error when evidence of a discrepancy between the actual state and the predicted or expected state rises above some threshold. In order to avoid false positive prediction-error signals, this threshold should be greater than the level of noise in the system. So the threshold for signalling a prediction error should be raised or relaxed if the level of noise in the system increases. This general principle is relevant to information processing in patients following brain injury (see Preston et al., 2010; Preston and Newport, 2014) but Jenkinson and Preston (2015, pp. 439–440) appeal to the same principle in the context of increased complexity of neural information processing.

If objects seen in a frontal mirror are mapped into the space in front of the mirror then visual processing of objects seen in a frontal mirror involves an additional spatial transformation, by comparison with visual processing of objects viewed directly – or through a clear glass window. If this transformation creates additional noise in the system then thresholds for prediction-error signalling might be relaxed and small discrepancies – for example, in the location, orientation, posture or appearance of a seen hand – might go unnoticed. Such discrepancies between the prosthetic hand and the participant's real hand would, normally, reduce the strength of the illusion of ownership but feelings of ownership in the frontal-mirror-view condition might be more likely to tolerate them (also see Hasegawa et al., 2020).

Supplementary Material

Discussion

D4. Frontal and parasagittal mirrors

Some RHI studies that have used a frontal-mirror view have also included what we shall call an *apparent-made-real* view. This is a condition in which, without a mirror, the rubber hand is presented in a *real* position and orientation corresponding to its *apparent* position and orientation when seen in a frontal mirror. For example, a participant seated at a testing table sees a confederate (or mannequin) seated on the opposite side of the table from them, with the rubber hand positioned in front of the confederate and oriented allocentrically, though egocentrically for the confederate (Bertamini et al., 2011, Experiment 4). Under such a condition, with no mirror and a third-person perspective (i.e., *apparent-made-real* view), the RHI is abolished (also see Preston et al., 2015). Would the RHI elicited with the parasagittal-mirror view – like the RHI elicited with the frontal-mirror view – be abolished in an apparent-made-real view?

To understand what the apparent-made-real view would involve, consider first the 15 cm + 30 cm and 15 cm + 45 cm asymmetrical conditions in our Experiment 2. The apparent position of the prosthetic hand is 15 cm behind the parasagittal mirror (i.e., 30 cm to the left of the participant's body midline), while the participant's hidden hand is 15 cm or 30 cm further to the left (see Figures 1D, E). In our study, the RHI was elicited in these distance conditions. In an apparent-made-real view, the directly viewed prosthetic hand would be 30 cm left of midline and the participant's hidden hand would be 15 cm or 30 cm further to the left behind an opaque divider. Would the RHI be abolished under those conditions? It seems unlikely that it would be abolished (cf. Preston, 2013, p. 179, Figure 1(a): prosthetic hand 17.5 cm left of midline, participant's hidden hand 35 cm further left, and Figure 1(c): prosthetic hand 35 cm left of midline, participant's hidden hand 17.5 cm further left), but experiments using both the Parasagittal-Mirror-RHI paradigm and the Classic-RHI paradigm would be needed to settle the question.

The possibility that the Parasagittal-Mirror-RHI and Frontal-Mirror RHI paradigms may differ in whether the RHI is abolished in the corresponding apparent-made-real view suggests that many similarities and differences between parasagittal- and frontal-mirror viewing are not yet fully understood.

Supplementary References

Hasegawa, H., Okamoto, S., Itoh, K., Hara, M., Kanayama, N., and Yamada, Y. (2020). Self-body recognition through a mirror: Easing spatial-consistency requirements for rubber hand illusion. *Psych*, *2*, 114–127. doi: 10.3390/psych2020011

Jenkinson, P. M., and Preston, C. (2017). The 'not-so-strange' body in the mirror: A principal components analysis of direct and mirror self-observation. *Consciousness and Cognition*, *48*, 262–272. doi: 10.1016/j.concog.2016.12.007

Preston, C., Jenkinson, P. M., and Newport, R. (2010). Anosognosia for hemiplegia as a global deficit in motor awareness: Evidence from the non-paralysed limb. *Neuropsychologia*, *48*, 3443–3450. doi: 10.1016/j.neuropsychologia.2010.07.027

Preston, C., Kuper-Smith, B. J., and Ehrsson, H. H. (2015). Owning the body in the mirror: The effect of visual perspective and mirror view on the full-body illusion. *Scientific Reports*, *5*:18345. doi: 10.1038/srep18345

Preston, C., and Newport, R. (2014). Noisy visual feedback training impairs detection of self-generated movement error: Implications for anosognosia for hemiplegia. *Frontiers in Human Neuroscience*, *8*:456. doi: 10.3389/fnhum.2014.00456

White, R. C., and Aimola Davies, A. M. (2015). The *no*-touch rubber hand paradigm and mirror-touch sensation: Support for the self-other theory of mirror-touch synesthesia. *Cognitive Neuroscience*, *6*, 146–147. doi: 10.1080/17588928.2015.1057483
